# Gamma and vega hedging using deep distributional reinforcement learning

**DOI:** 10.3389/frai.2023.1129370

**Published:** 2023-02-22

**Authors:** Jay Cao, Jacky Chen, Soroush Farghadani, John Hull, Zissis Poulos, Zeyu Wang, Jun Yuan

**Affiliations:** ^1^Joseph L. Rotman School of Management, University of Toronto, Toronto, ON, Canada; ^2^Department of Computer Science, University of Toronto, Toronto, ON, Canada

**Keywords:** derivatives, hedging, gamma, vega, delta-neutral, reinforcement learning, D4PG, quantile regression

## Abstract

We show how reinforcement learning can be used in conjunction with quantile regression to develop a hedging strategy for a trader responsible for derivatives that arrive stochastically and depend on a single underlying asset. We assume that the trader makes the portfolio delta-neutral at the end of each day by taking a position in the underlying asset. We focus on how trades in options can be used to manage gamma and vega. The option trades are subject to transaction costs. We consider three different objective functions. We reach conclusions on how the optimal hedging strategy depends on the trader's objective function, the level of transaction costs, and the maturity of the options used for hedging. We also investigate the robustness of the hedging strategy to the process assumed for the underlying asset.

## 1. Introduction

Hedging a portfolio of options or more complex derivative securities involves sequential decision-making. The position that is taken to reduce risk must be revised daily, or even more frequently, to reflect movements in market variables. Reinforcement learning (RL) is therefore an ideal tool for finding a hedging strategy that optimizes a particular objective function.

Previous studies concerned with the application of RL to hedging decisions include Halperin (2017), Buehler et al. (2019), Kolm and Ritter (2019), and Cao et al. (2021). These authors consider how RL can be used to hedge a single call or put option using a position in the underlying asset. The key measure of risk they consider is the delta of the option. This is the first partial derivative of the option price with respect to the price of the underlying asset and is, therefore, a measure of exposure to small changes in the price of the underlying asset. The position taken in the underlying asset can reduce or eliminate this exposure.

In practice, a trader responsible for derivatives dependent on a particular underlying asset does not have a great deal of discretion about delta hedging: the trader is usually required to eliminate, or almost eliminate, the delta exposure each day.^1^ A more interesting decision that the trader has to make concerns the gamma and vega exposure. Gamma is the second partial derivative of the value of the portfolio with respect to the underlying asset and is a measure of exposure to large asset price changes. Vega is the partial derivative of the value of the portfolio with respect to the volatility of the asset price and is a measure of exposure to volatility changes. A trader is typically subject to limits on the permissible size of a portfolio's gamma and vega exposure but has discretion on how gamma and vega are managed within those limits. Whereas, delta can be changed by taking a position in the underlying asset, gamma and vega can be changed only by taking a position in an option or other derivative.

In deciding on a hedging strategy, traders must take transaction costs into account. These arise because of the difference between the bid price (the price at which a financial instrument can be sold to a market maker) and the ask price (the price at which the financial instrument can be bought from the market maker). The mid-market price (i.e., the average of the bid and ask prices) is usually considered to be the true value of the instrument. As a result, the transaction cost can be quantified as half the difference between the bid and ask prices. Transaction costs are typically quite small for trading the underlying asset but much larger for trading options and other derivatives.

Rather than considering a single option, we consider a portfolio of options that evolves stochastically. We assume that the portfolio is made delta-neutral at the end of each day. Before a final trade in the underlying asset is made to achieve delta neutrality, the trader makes a decision on how gamma and vega are managed. As a simplification, we assume that there are no transaction costs associated with trading the underlying asset (As mentioned earlier, these costs are usually quite small.). Alternative levels for transaction costs for the options brought into the portfolio for hedging are considered.

Our article can be regarded as a “proof of concept”. We make relatively simple assumptions about the process driving the arrival of client options, the options available for hedging, the process driving the price of the underlying asset, the nature of transaction costs, and the trader's objective function. The assumptions appropriate to a particular trading situation are likely to be more complicated than those we make. Our objective is to illustrate how RL can be used to develop a strategy for managing gamma and vega risk. The same reinforcement approach can be used in all situations, but the strategies resulting from applications of the approach are likely to depend on the assumptions made.

The assumptions we make to produce illustrative results are as follows. Client options arrive according to a Poisson process with an intensity of 1.0 per day. The options entered into by clients have 60 days to maturity and are equally likely to be long or short. We assume that a single option is available for hedging. This is an at-the-money call with a particular time to maturity.^2^ Each day, the trader chooses the position (if any) to be taken in the option. The trader then trades the underlying asset to make the whole portfolio (options entered into with clients plus options entered into for hedging) delta-neutral. The transaction costs associated with an option trade are assumed to be a specified proportion of the value of that trade. The performance of the hedging strategy is considered over 30 days. At the end of that period, all options in the portfolio are valued at their mid-market prices.

The choice of an objective function depends on the risk preferences of the trader. We consider three different objective functions. The first involves a trade-off between the mean and standard deviation of the trader's profit/loss over a predetermined period. This objective function has been used by other researchers as a measure of the efficiency of hedging but it can be criticized because it penalizes good as well as bad outcomes. We, therefore, also consider two objective functions that focus only on losses. These are value at risk (VaR) with a 95% confidence level and conditional value at risk (CVaR; also referred to as expected shortfall) with a 95% confidence level. As is well-known, CVaR, although more complicated than VaR, has better theoretical properties. In the case of the first objective function, we set the objective (to be minimized) as the mean loss over the period considered plus 1.645 times the standard deviation of the loss (with gains being considered negative losses). This is an interesting choice because, if the distribution of the profit/loss after hedging was normal, it would be equivalent to the second (VaR) objective function.

In our experiments, all the options in the portfolio at any given time are “plain vanilla” European call options, and the process assumed for the underlying asset does not include jumps. As a result, the distribution of the change in the value of the hedged portfolio between hedging decisions is approximately normal and the differences between the hedging strategies for the three objective functions we consider are relatively small. When other options such as barrier options and digital options are considered and when more elaborate processes for the underlying asset are considered, the change in the value of the hedged portfolio between hedge rebalancing is liable to have a non-symmetrical distribution and bigger differences between the results obtained for the three objective functions can be expected.

Our approach can be used to assess the profitability of trading derivatives when a particular hedging strategy is used. The expected profit on client options (i.e., the expected transaction costs earned on these options) can be compared with the expected loss on hedging (i.e., the expected transaction costs paid on the options used for hedging). Assuming the former is calculated in the same way as the latter, we find that the hedging strategies for the cases we consider are economically feasible in the sense that expected transaction costs earned are greater than those paid.

The rest of the article is organized as follows. Section 2 describes the RL model we use. Section 3 explains the models we use for the evolution of the underlying asset price. Section 4 presents our illustrative results. It first focuses on gamma hedging by using a model where volatility is constant and then considers a stochastic volatility world where both gamma and vega have to be monitored. Section 5 investigates the robustness of the RL strategies to the assumptions made about the asset price process. Conclusions and suggestions for further study are discussed in the final section.

## 2. The RL model

In the standard RL formulation, the goal of the agent is to maximize expected future rewards. Specifically, the agent attempts to maximize at time *t* the expected value of *G*_*t*_, where


$$\begin{array}{l} G_{t}=R_{t+1}+\gamma R_{t+2}+\gamma^{2}R_{t+3}+\ldots +\gamma^{T-1}R_{T} \end{array}$$

where *R*_*t*_ is the reward at time *t*, *T* is a horizon date, and γ ∈ (0, 1] is a discount factor. *Q*(*S, A*) is defined as the expected value of *G*_*t*_ from taking action *A* in state *S* at time *t*. The standard RL formulation involves maximizing *Q*(*S, A*). This is not appropriate for our application (and many other applications in finance) because we are concerned with exploring risk-return trade-offs. A class of RL methods collectively referred to risk-aware RL has recently been developed to extend RL so that other attributes of the distribution of *G*_*t*_ besides the mean can be considered.

Tamar et al. (2016) showed how both the first and second moments (and possibly higher moments) of the distribution of *G*_*t*_ can be updated using more than one *Q*-function. Cao et al. (2021) used this approach and produced results for an objective function involving the mean and standard deviation of *G*_*t*_, where one *Q*-function approximates the mean of the terminal return and a second *Q*-function approximates the expected value of the square of the terminal return. However, the approach is less than ideal. It is computationally demanding and imposes restrictions on the objective functions that can be considered.

Bellemare et al. (2017) pointed out that the Bellman equation used to update *Q*(*S, A*) can also be used to update distributions. *Z*(*S, A*) is defined as the distribution of *G*_*t*_ resulting from action *A* in state *S*. They suggest a procedure known as C51 where *Z*(*S, A*) is defined as a categorical distribution and probabilities are associated with 51 fixed values of *G*_*t*_. As new points on the distribution are determined in trials, they are allocated to the neighboring fixed points. Barth-Maron et al. (2018) incorporate this approximation method into an actor-critic RL model, where one neural network (NN; critic) estimates the categorical distribution *Z*(*S, A*) and another (NN; actor) decides the actions that the agent takes based on information from the critic network. These two NNs are trained simultaneously. Their framework also supports the use of multiple agents at the same time for distributed exploration of the search space in large-scale RL problems. The overall process is known as Distributed Distributional Deep Deterministic Policy Gradients (D4PG) and has become an impactful RL algorithm with applications primarily in robotic control systems.

Dabney et al. (2018) proposed an alternative distributional RL algorithm, QR-DQN, that uses quantile regression (QR) to approximate *Z*(*S, A*). The distribution is represented with a discrete set of quantiles whose positions are adjusted during training. Compared to C51, QR generalizes better and is more flexible. It can efficiently approximate a wide range of distributions without the need to specify fixed points *a priori*. The QR procedure is wrapped within Deep Q-Learning (DQN), a well-known and studied RL algorithm. Unlike actor-critic architectures, DQN uses only a single NN. The NN approximates *Z*(*S, A*) and the agent actions are generated using a greedy algorithm. The authors show that QR produces better results than C51 when both are used in DQN algorithms and applied to Atari 2600 games.

For the hedging problem at hand, QR is an attractive solution as it is straightforward to measure VaR and CVaR directly on the quantile-based representation that it generates. In contrast, C51 requires interpolating on the categorical distribution to obtain quantiles, which induces additional approximation errors. Furthermore, an actor-critic architecture similar to the one used in D4PG is also more desirable compared to DQN. The latter is a lightweight sample-efficient model, often used in cases where simulation is slow. In our setting, simulating the portfolio is a fast process and not the bottleneck, so actor-critic models can handle the task well while exploring the search space rigorously. We posit that this is an important step toward identifying hedging strategies that are sensitive to different hedging scenarios and volatility movements in the underlying.

The combination of QR and D4PG has not been explored in the literature. In this study, as one of our contributions, we modify D4PG to support QR at the output of the critic NN. The resulting algorithm, which we refer to as D4PG-QR, is used for all experiments discussed later. We observed that our implementation is superior to D4PG (which uses C51 by default) for VaR and CVaR objective functions and as good as D4PG for the mean/standard deviation objective in terms of accuracy and computational efficiency.^3^

We now describe how we use the distributional RL framework for hedging. We assume that we are hedging a portfolio of client options. The portfolio composition evolves as new client options arrive. Arrivals are modeled with a Poisson process, the intensity of which can be specified as a parameter in our framework. In expectation, half of the client orders are short and half is long. At the time of each rebalancing, we use an at-the-money call option for hedging. We assume that the trader rebalances at time intervals of *t*.

Prices for the underlying asset are generated by a pre-specified stochastic process, which will be described in the next section. All options are assumed to give the holder the right to buy or sell 100 units of the underlying asset. We set *t* equal to 1 day and the time period, *T*, considered is 30 days. We set γ = 1 as this period is fairly short.

The state at time *i*Δ*t* is defined as follows:

- The price of the underlying asset.
- The gamma of the portfolio.
- The vega of the portfolio.
- The gamma of the at-the-money option used for hedging.
- The vega of the at-the-money option used for hedging.

The portfolio gamma is calculated as the sum of the gammas of all the options in the portfolio. Portfolio vega is calculated similarly.

The action at time *iΔt* is the proportion of maximum hedging that is done. Specifically, we do not allow the agent to try any arbitrary position in the hedging option during training. Instead, we determine at each time step the maximum hedge permitted such that at least one of the two following criteria is satisfied: (a) the ratio of gamma after hedging to gamma before hedging falls in the range [0,1] and (b) the ratio of vega after hedging to vega before hedging falls in the range [0,1]. The action of the agent is constrained to lie within the resulting range.^4^ This ensures that the agent is hedging rather than speculating. Restricting the action space increases sample efficiency and improves convergence. We avoid wasteful simulations where the agent tries actions that are known not to be part of any optimal hedging strategy and, therefore, reduce the size of the search space.

The definitions we use are:

*V*_*i*_: The value of the option used for hedging at time *iΔt*.
*H*_*i*_: The position taken in the option used for hedging at time *iΔt*.
κ: The transaction cost associated with the option used for hedging as a proportion of the value of the option.
*P*_*i*_: The total value of options in the portfolio at time *iΔt* that have not previously expired.

The variable *P*_*i*_ includes all the options in the portfolio that have not expired before time *iΔt*. If an option expires at time *iΔt*, its value at time *iΔt* is set equal to its intrinsic value.

The reward at time *iΔt* (*i* > 0) is therefore^5^


$$\begin{array}{l} R_{i}=-\kappa |V_{i}H_{i}|+\left(P_{i}-P_{i-1}\right) \end{array}$$

### 2.1. Hedging with D4PG-QR

**Figure 1:**
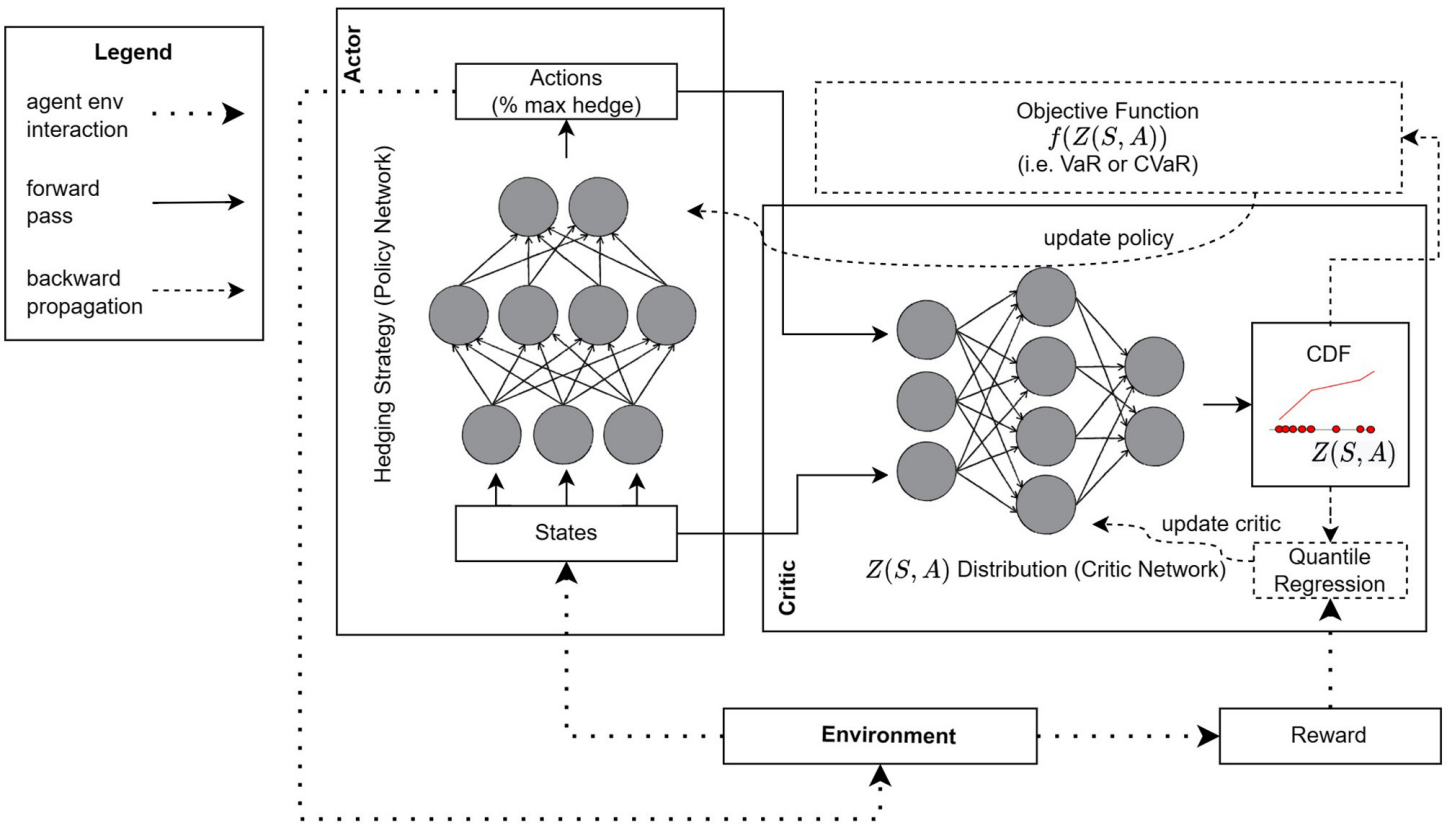
RL architecture for actor-critic learning and proposed networks.

The RL framework is provided in Figure 1, and it includes the proposed D4PG-QR architecture. There are three major components, namely, the environment, an actor NN, and a critic NN. The trading environment involves a simulator of how the portfolio composition and value evolve over time based on the hedging positions that the agent takes, the market dynamics for the underlying, and the client option arrival process. At any given time, the simulator computes the next state and the reward. The actor NN (also known as the policy network) implements the hedging strategy. It takes as input a state and outputs the position of the hedger. The critic NN takes as inputs a state, *S*, and the action from the actor's output, *A*. Its role is to (a) estimate the distribution of the trading loss at the end of the hedging period, *Z*(*S, A*), when taking action *A* in state *S*, and (b) compute gradients that minimize the objective function *f*(*Z*(*S, A*)). These gradients are then used by the actor NN to improve the actions that it outputs in subsequent rounds. For the actor NN, we used three layers with 256 neurons per layer. For the critic NN, we used three layers with 512 neurons per layer for the first two layers and 256 neurons for the final layer.

The key difference between D4PG and our architecture is that *Z*(*S, A*) is represented by a set of discrete quantiles, whose probabilities are fixed but their position is adjusted during training based on the rewards observed over several episodes. We used 1% quantile intervals so that the probability distribution is represented by 100 points. This quantile-based representation also allows us to compute the CDF of *Z* (*S, A*). The adjustment is done *via* QR using the quantile Huber loss as a loss function. The Huber loss function is defined as follows:


$$\begin{array}{l} L_{k}\left(u\right)=\{\begin{array}{c} \frac{1}{2}u^{2},\;|u|\leq k\\ k\left(\left|u\right|-\frac{1}{2}k\right),\;otherwise \end{array} \end{array}$$

The quantile Huber loss used as the critic's loss in D4PG-QR corresponds to an asymmetric squared loss in the interval [−*k, k*] and acts as a regular quantile loss outside that interval. Specifically, the critic's quantile Huber loss for any quantile τ ∈ [0, 1] and parameter *k* is defined as follows:


$$\begin{array}{l} h_{\tau }^{k}\left(u\right)=|\tau -\delta_{u<0}|\;L_{k}\left(u\right) \end{array}$$

where δ_*u* < 0_ denotes a Dirac at all negative points.

Gradients that minimize the loss are used to improve the critic's accuracy when estimating *Z*(*S, A*). In the early training stages, the critic's estimate of *Z*(*S, A*) is very rough and thus the actor's hedging policy is far from optimal. As the critic improves so does the actor's policy to the point where both NNs converge to some local optimum: the critic ends up with a good estimate of the distribution of hedging loss for a large range of states and actions and the actor learns a good policy that optimizes the corresponding objective for any state that it is presented with.

We should point out that there are several other components (not shown in Figure 1) that we borrow from the D4PG algorithm, the most important being the use of a replay buffer. With a replay buffer, instead of learning a policy using state transitions in the order that they appear based on the actor's actions, we simulate and store a large set of state transitions (along with their rewards) in a table. Then, to update the critic and actor NNs, we sample transitions and their rewards uniformly at random from that table. This aids with the convergence of the NNs for two reasons. First, the samples are i.i.d. which is beneficial when updating NNs in general. Second, the replay buffer allows for “experience replay”. That is, we can use the same state transition and reward multiple times when updating the critic and actor. Since the improvements in estimating *Z*(*S, A*) are incremental, applying gradient updates several times over the same state transitions leads to faster convergence. The pseudocode describing the parameter update process for the actor and critic networks in D4PG-QR is given in Algorithm 1.

**Algorithm 1:** D4PG-QR.

**Input**: batch size *N*, quantile representation ${\;Z}_{w}^{\tau_{j}}$ of *Z*_*w*_for *j* = 1… *M*, initial learning
rates α_0_ (actor, π_θ_) and β_0_ (critic, *Z*_*w*_)
1: Initialize actor parameters θ and critic
parameters *w* at random
2: **for** *t* = 1:*T* **do**
3: Sample *N* transitions (*S*_*i*_, *A*_*i*_, *R*_*i*_) → (*S*_*i*+1_, *A*_*i*+1_, *R*_*i*+1_)
from replay buffer
4: Compute target distributions
*Y*_*i*_←*R*_*i*_+γ*Z*_*w*_(*S*_*i*+1_, π_θ_(*S*_*i*+1_))
5: Compute the expected quantile Huber loss
$L_{i}\left(w\right)\leftarrow \overset{M}{\underset{j=1}{\sum }}E_{Y_{i}}\left[h_{\tau_{j}}^{k}\left(Y_{i}-Z_{w}^{\tau_{j}}\left(S_{i},A_{i}\right)\right)\right]$
6: Compute actor and critic updates:
7: $\Delta_{w}\leftarrow \frac{1}{N}\overset{N}{\underset{i=1}{\sum }}\nabla_{w}L_{i}\left(w\right)$
8: $\Delta_{\theta }\leftarrow \frac{1}{N}\overset{N}{\underset{i=1}{\sum }}\nabla_{\theta }\pi_{\theta }\left(S_{i}\right){E\left[\nabla_{A}f\left(Z_{w}\left(S_{i},A\right)\right)\right]|}_{A=\pi_{\theta }\left(S_{i}\right)}$
9: *w*←*w*+β_*t*_Δ_*w*_
10: θ←θ+α_*t*_Δ_θ_

## 3. The asset pricing model

We assume that the risk-neutral behavior of the underlying asset price, *S*, and its volatility, σ, is governed by the following stochastic processes^6^:


$$\begin{array}{lll} dS & = & \left(r-q\right)Sdt+\sigma Sdz_{1}\\ d\sigma & = & v\sigma dz_{2} \end{array}$$

where *dz*_1_ and *dz*_2_ are Wiener processes with constant correlation ρ. The volatility of the volatility variable, *v*, the risk-free rate, *r*, and the dividend yield, *q*, is assumed to be constant. We assume that the real-world expected return μ is also constant. The real-world process for the asset price is the same as that given above with *r*−*q* replaced by μ.

This model is a particular case of the SABR model developed by Hagan et al. (2002).^7^ It has the attractive property that there is a good analytic approximation to a European option's implied volatility. σ_0_ and *S*_0_ are defined as the initial values of σ and *S*, respectively. If the strike price and time to maturity of a European call option are *K* and *T*, respectively, the estimate of the implied volatility is σ_0_*B* when *F*_0_ = *K* and $\frac{\sigma_{0}B\varphi }{\chi }$, otherwise, where


$$\begin{array}{lll} F_{0} & = & S_{0}e^{\left(r-q\right)T}\\ B & = & 1+\left(\frac{\rho v\sigma_{0}}{4}+\frac{\left(2-3\rho^{2}\right)v^{2}}{24}\right)T\\ \phi & = & \frac{v}{\sigma_{0}}\ln\left(\frac{F_{0}}{K}\right)\\ \chi & = & \ln\left(\frac{\sqrt{1-2\rho \varphi +\phi^{2}}+\phi -\rho }{1-\rho }\right) \end{array}$$

Denoting the implied volatility by σ_*imp*_, the value of the option is given by the Black–Scholes–Merton formula as follows:


(1) $$\begin{array}{l} S_{0}N\left(d_{1}\right)e^{-qT}-Ke^{-rT}N\left(d_{2}\right) \end{array}$$

where


$$\begin{array}{lll} d_{1} & = & \frac{\ln\left(\frac{S_{0}}{K}\right)+\left(r-q+\frac{\sigma_{imp}^{2}}{2}\right)T}{\sigma_{imp}\sqrt{T}}\\ d_{2} & = & d_{1}-\sigma_{imp}\sqrt{T} \end{array}$$

and *N* is the cumulative normal distribution function. When *v* = 0, the implied volatility is constant and equal to σ_0_, and the SABR model reduces to the option pricing model developed by Black and Scholes (1973) and Merton (1973).

As mentioned, delta is the first partial derivative of the option price with respect to the asset price, gamma is the second partial derivative with respect to the asset price, and vega is the first partial derivative with respect to volatility. Under the model that is being assumed, a natural idea is to regard a European option value as a function of *S* and σ for the calculation of these partial derivatives. However, the usual practitioner approach is to regard the option value as a function of *S* and σ_*imp*_ when Greek letters are calculated. This is the approach we will adopt. Denoting delta, gamma, and vega by Δ, Γ, and Υ, respectively, Equation (1) gives the following formula:


$$\begin{array}{lll} \Delta & = & N\left(d_{1}\right)e^{-qT}\\ \Gamma & = & \frac{N^{\prime }\left(d_{1}\right)e^{-qT}}{S_{0}\sigma_{imp}\sqrt{T}}\\ \Upsilon & = & S_{0}e^{-qT}\sqrt{T}N^{\prime }\left(d_{1}\right) \end{array}$$

The delta, gamma, and vega of a portfolio are calculated by summing those for the individual options in the portfolio.

## 4. Results

We conducted the hedging experiment with three different objective functions. The objective functions are calculated from the total gain/loss during the (30-day) period considered. The first objective function to be minimized is


$$\begin{array}{l} m+1.645s \end{array}$$

where *m* is the mean of the loss and *s* is its standard deviation.^8^ If the distribution of losses was normal, this objective function would minimize the 95^th^ percentile of the portfolio loss distribution.

The second and third objective functions minimize the VaR and CVaR. For both measures, we use a 95% confidence level. Our VaR measure (VaR95) is, therefore, the 95^th^ percentile of the portfolio loss, while our CVaR measure (CVaR95) is the expected loss conditional on the loss being worse than the 95^th^ percentile of the loss distribution.

We report the results of over 5,000 test scenarios for a 30-day hedging period on average. The test scenarios are different from the (much greater number of) scenarios on which agents are trained. Within each set of tests, the scenarios were kept the same.

### 4.1. Hedge ratios

The gamma (vega) hedge ratio when a hedging decision is made is the proportional amount by which gamma (vega) is reduced. It is defined as one minus the gamma (vega) exposure of the portfolio after the hedging divided by the gamma (vega) exposure of the portfolio before hedging, i.e.,


$$\begin{array}{l} \text{Gamma Hedge Ratio}=1-\frac{\underset{t}{\sum }sign\left(\Gamma^{t}\right)\Gamma^{t^{+}}}{\underset{t}{\sum }sign\left(\Gamma^{t}\right)\Gamma^{t}}\\ \text{Vega Hedge Ratio}=1-\frac{\underset{t}{\sum }sign\left(\Upsilon^{t}\right)\Upsilon^{t^{+}}}{\underset{t}{\sum }sign\left(\Upsilon^{t}\right)\Upsilon^{t}} \end{array}$$

where Γ^*t*^ is portfolio gamma before hedging at time *t* and Γ^*t*^^+^ is the ratio after hedging. Similarly, Υ^*t*^ and Υ^*t*^^+^are the portfolio vega before and after hedging. We report the average values of the gamma and vega hedge ratios across all test scenarios and all hedging actions.

### 4.2. Gamma hedging results

We start by focusing on gamma risk by setting *v* = 0 so that the SABR model reduces to the Black–Scholes–Merton model and there is no vega risk. We remove the two vega-related state variables in this experiment as they are not relevant to the agent. As already mentioned, we assume that the RL agent has to hedge the client orders arriving according to the Poisson process with an intensity of 1.0 per day. Each client order is assumed to be for a 60-day option on 100 units of the underlying assets and has an equal probability of being long or short. We run experiments with transaction costs assumed to be 0.5, 1, and 2% of the option price. A 30-day at-the-money option is used as the hedging option. The initial stock price is set at $10 and the volatility is 30% per annum.^9^

Table 1 compares the performance of RL agents with delta-neutral and delta-gamma-neutral strategies when they use the three different objective functions and the three different transaction cost assumptions. It shows that RL improves the hedger's objective functions compared to the simpler strategies.^10^ The outperformance of the RL agents can be attributed to their ability to adjust their hedging policies for different transaction costs. As the transaction cost increases, the RL agents reduce the amount of gamma they are hedging. The expected cost of hedging increases as the transaction cost increases. However, because less hedging is done, the expected cost of hedging rises more slowly than transaction costs.

**Table 1:** Results of tests when volatility is constant.

	**Objective function value for**			**RL gamma hedge ratio**	**Expected RL transaction cost**
**Objective function**	**Delta**	**Delta-gamma**	**RL**		
**0.5% transaction cost**					
Mean-Std	24.61	5.78	5.44	0.83	3.00
VaR95	24.29	5.78	5.47	0.75	2.80
CVaR95	36.64	7.13	6.78	0.79	2.92
**1% transaction cost**					
Mean-Std	24.61	9.93	8.36	0.57	4.58
VaR95	24.29	10.12	8.63	0.56	4.51
CVaR95	36.64	11.55	10.02	0.60	4.71
**2% transaction cost**					
Mean-STD	24.61	18.74	12.73	0.30	5.91
VaR95	24.29	19.11	13.05	0.24	5.15
CVaR95	36.64	21.10	15.37	0.29	5.78

The Delta column shows the values of the objective functions when only delta hedging is carried out; the Delta-Gamma column shows the values when delta and gamma are fully hedged. The RL results column shows the values when RL agents are used to minimize the objective functions in the first column. The final two columns report the average percentage of gamma hedged by the RL agent and the expected loss arising from transaction costs from the trading conducted by the RL agent.

The value of one client option is ~$60 based on Black–Scholes pricing formula with our chosen parameters. With the expected arrival of one option per day, the expected profit from client options over 30 days is, therefore, ~1,800 times the premium over the mid-market value charged on client options. If this premium is κ times the option price (i.e., the transaction cost faced by clients of the dealer is the same as the transaction cost faced by the dealer when hedging), the results in Table 1 show that the cost of hedging is comfortably covered by the transaction costs earned on client options. For example, when κ* is* 1%, the expected cost of the RL hedge is <$5, whereas the expected profit from client options is $18.

**Figure 2:**
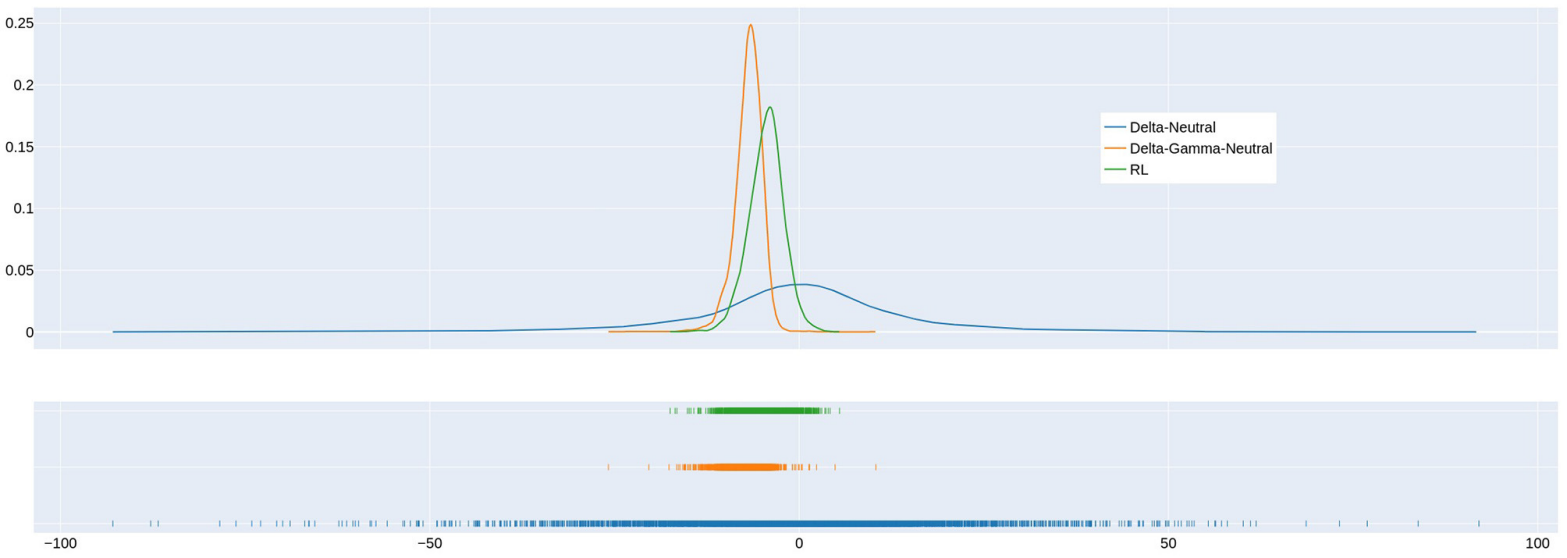
Comparison of gain distribution for delta hedging, delta-gamma-neutral hedging, and hedging by the VaR95 RL agent when transaction cost = 1%. Note that the 5^th^ percentile of the gain distribution corresponds to the 95th percentile loss in Table 1.

Figure 2 shows the distribution of the gain for the delta, delta-gamma, and RL strategies for the VaR95 agent when transaction costs are 1%. An examination of the left tails of the distributions in the rug plot shows that the VaR95 RL strategy has a smaller probability of experiencing large losses than the delta and delta-gamma strategies. The expected cost of delta hedging is zero (This is as expected because we assume no transaction costs for delta hedging.). The expected cost of RL is clearly less than the delta-gamma strategy.

### 4.3. Risk limits

Traders are usually subject to limits on their gamma and vega exposure at the end of each day. These limits can easily be incorporated into RL by imposing constraints, in addition to those mentioned in Section 2, on the range of hedging transactions that are considered.

As an experiment, we compared RL with delta-gamma hedging when gamma is reduced only when the exposure exceeds a risk limit. We choose a risk limit equal to 10% of the max gamma exposure of the unhedged portfolio in the simulated environment, which could be an appropriate choice for a trading desk to set the risk limit in practice. As Table 2 shows, the RL agent continues to outperform the rule-based agents across different objective functions and transaction costs.

**Table 2:** Results of tests when volatility is constant.

	**Objective function value for**	
**Objective function**	**Delta-gamma**	**RL**
**0.5% transaction cost**		
Mean-Std	7.27	6.80
VaR95	7.40	6.43
CVaR95	9.20	7.84
**1.0% transaction cost**		
Mean-Std	9.59	9.19
VaR95	9.69	8.86
CVaR95	11.62	10.39
**2.0% transaction cost**		
Mean-Std	14.85	13.34
VaR95	15.17	13.29
CVaR95	17.44	15.33

The Delta-Gamma agent shows the values when delta is fully hedged, and gamma is fully hedged every time the dollar gamma exposure is >50. The RL results show the values when delta is fully hedged and gamma exposure is hedged according to the VaR95 RL agent policy when the dollar gamma exposure >50.

### 4.4. Impact of volatility uncertainty

We now move on to consider situations where volatility is uncertain by setting σ_0_ equal to 30%, *v* equal to 0.3, and ρ = −0.7 in the SABR model described in Section 3. Other parameters and assumptions are described in Section 4.2. With two options, both gamma and vega could be completely neutralized. However, we assume that only a single at-the-money option is available. Whereas, short maturity at-the-money options are most useful for hedging gamma, longer maturity options work better for vega. Table 3 illustrates this by showing results for the following two different maturities of the options used for hedging: 30 and 90 days. As before, three different transaction costs, as a percent of the hedging option price, are considered. The hedging policy of the RL agents is adaptive to the maturity of the hedging options. The performance of the RL agents is closest to the delta-gamma-neutral policy when short-dated maturity options are used for hedging and the performance moves closer to the delta-vega neutral policy as the option maturity increases. Similar to the results when the Black–Scholes–Merton model is used, the RL agents reduce the amount of gamma and vega hedging as transaction cost increases. As in the constant volatility example, it can be shown that the expected cost of hedging paid is comfortably covered by the transaction costs earned on client options in the situations we consider.

**Table 3:** Results of tests when volatility is stochastic.

**Hedge option** **maturity**	**Objective function**	**Value of objective function for**				**RL** **gamma hedge ratio**	**RL vega hedge ratio**	**Expected RL transaction cost**
		**Delta**	**Delta-gamma**	**Delta-vega**	**RL**			
**Transaction costs** = **0.5%**								
30 days	Mean-Std	35.76	19.46	44.82	17.76	0.53	0.17	2.75
	VaR95	34.43	19.23	42.90	19.31	0.61	0.18	3.25
	CVar95	52.91	27.53	62.77	26.06	0.73	0.16	3.44
90 days	Mean-Std	35.76	25.25	15.47	14.28	0.27	0.55	4.90
	VaR95	34.43	24.43	15.40	14.41	0.24	0.47	4.53
	CVar95	52.91	31.96	20.21	18.17	0.26	0.63	5.04
**Transaction costs** = **1%**								
30 days	Mean-Std	35.76	23.06	51.36	20.03	0.50	0.13	4.40
	VaR95	34.43	23.02	50.24	20.22	0.45	0.12	3.98
	CVar95	52.91	31.55	69.92	26.81	0.42	0.10	4.00
90 days	Mean-Std	35.76	35.29	22.20	18.61	0.14	0.37	6.21
	VaR95	34.43	35.01	22.05	18.86	0.15	0.32	6.36
	CVar95	52.91	42.63	27.18	24.58	0.12	0.31	5.65
**Transaction costs** = **2%**								
30 days	Mean-Std	35.76	30.51	64.77	24.17	0.33	0.11	7.01
	VaR95	34.43	30.67	64.56	23.85	0.29	0.07	5.70
	CVar95	52.91	39.79	84.38	31.57	0.27	0.07	5.62
90 days	Mean-Std	35.76	56.67	36.58	25.20	0.09	0.21	9.31
	VaR95	34.43	56.97	36.78	25.73	0.08	0.16	7.80
	CVar95	52.91	65.05	42.14	32.62	0.09	0.16	8.13

The Delta-column shows the values of the objective function when only delta hedging is carried out; the Delta-Gamma column shows the values when delta and gamma are fully hedged; the Delta-Vega column shows the values when delta and vega are fully hedged. The RL results column shows the values when RL agents are used to minimize objective functions. The final three columns report the averages of the gamma and vega hedged by RL and the expected RL loss from transaction costs.

**Figure 3:**
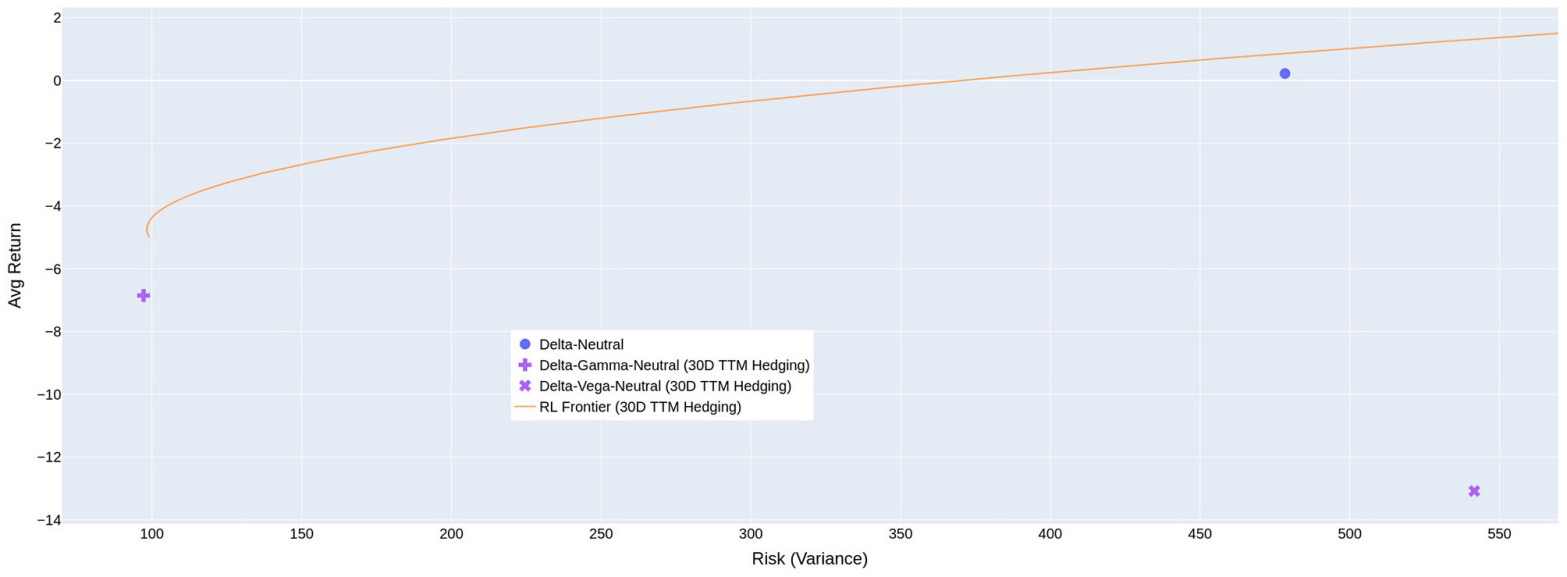
Risk-return trade-offs that are possible using RL with different objective functions. The positive average cost of hedging reported in previous tables means the average return of the agent is negative.

By training multiple RL agents with different VaR percentiles as objective functions and using a 30-day maturity hedging option, we have constructed a frontier to represent the risk and return for different levels of risk aversion when transaction cost is equal to 1%, as shown in Figure 3. The figure shows that the RL agents generally outperform simple rule-based strategies such as delta-neutral, delta-gamma-neutral, and delta-vega-neutral.

## 5. Robustness tests

Our tests, so far, have assumed that the agent correctly estimates the stochastic process followed by the underlying asset. We now consider the sensitivity of hedge performance to a stochastic process that is different from the one assumed in developing the hedging strategy. We assume that the true stochastic process is the one considered in Table 3 where the volatility of the volatility parameter, *v*, is 0.3 and the initial volatility σ is 30%. The option used for hedging is assumed to last for 90 days and the transaction costs are 1% of the option price.

Table 4 considers the situation where the agent develops the hedging strategy with the correct value of σ but with values of *v* equal to 0, 0.15, 0.3, 0.45, and 0.6. Table 5 considers the situation where the correct value of *v* is used but the hedging strategy is developed with values of σ equal to 10, 20, 30, 40, and 50%. Overall, the results indicate that hedging performance is fairly robust to the values assumed for the parameters. Interestingly, in both cases, there is virtually no deterioration in the performance of the hedge when the parameters used to determine the hedging strategy are too high, but a noticeable decrease in performance when they are too low. However, the expected cost of hedging is greater when the parameter estimates are too large.

**Table 4:** Values of the objective function and expected cost of hedging when the hedging strategy is developed with values of the volatility of volatility, *v*, that are different from the actual 0.3 value.

***v***	**Objective function**	**Value of objective function**	**Gamma hedge ratio**	**Vega hedge ratio**	**Expected transaction costs**
0.0	Mean-Std	23.29	0.17	0.16	6.03
	VaR95	23.96	0.18	0.16	6.43
	CVaR95	32.79	0.21	0.17	7.08
0.15	Mean-Std	21.49	0.15	0.17	6.13
	VaR95	20.98	0.11	0.18	4.99
	CVaR95	25.86	0.17	0.24	6.66
0.3	Mean-Std	18.61	0.14	0.37	6.21
	VaR95	18.86	0.15	0.32	6.36
	CVaR95	24.58	0.12	0.31	5.65
0.45	Mean-Std	18.72	0.19	0.48	7.60
	VaR95	18.73	0.16	0.41	7.08
	CVaR95	22.97	0.19	0.40	7.71
0.6	Mean-Std	19.66	0.20	0.62	9.06
	VaR95	18.47	0.17	0.48	7.46
	CVaR95	23.48	0.20	0.52	8.74

The initial volatility, σ, is assumed to be 30%.

**Table 5:** Values of the objective function when the hedging strategy is developed with values of the initial volatility, σ, that are different from the actual 30% value.

**σ**	**Objective function**	**Value of objective function**	**Gamma hedge ratio**	**Vega hedge ratio**	**Expected transaction costs**
10%	Mean-Std	26.57	0.28	0.44	11.12
	VaR95	28.58	0.29	0.34	10.40
	CVaR95	29.81	0.36	0.39	11.93
20%	Mean-Std	21.48	0.21	0.50	8.94
	VaR95	19.61	0.22	0.53	8.37
	CVaR95	27.07	0.18	0.46	7.79
30%	Mean-Std	18.61	0.14	0.37	6.21
	VaR95	18.86	0.15	0.32	6.36
	CVaR95	24.58	0.12	0.31	5.65
40%	Mean-Std	18.88	0.18	0.40	7.18
	VaR95	20.92	0.17	0.35	7.13
	CVaR95	23.38	0.15	0.35	6.64
50%	Mean-Std	19.95	0.12	0.32	6.02
	VaR95	19.62	0.14	0.40	7.13
	CVaR95	24.95	0.23	0.35	8.96

The volatility of volatility parameter, *v*, is assumed to be 0.3.

## 6. Conclusion and further study

We have illustrated how D4PG can be used in conjunction with QR to produce hedging strategies that are consistent with the objective of the hedger. Our approach allows the agent to take transaction costs into account when developing a policy for managing gamma and vega. Our results illustrate that the RL agent is able to find a good balance between (a) only hedging delta and (b) fully hedging gamma or vega as well as delta.

There are a number of ways in which our research can be extended. A straightforward extension is to explore how well the hedging strategies work for other stochastic volatility processes for the underlying asset and for processes where there are jumps in the price of the underlying asset. Alternatively, a joint process for the asset price and volatility surface, such as that proposed by Francois et al. (2023), can be investigated. The analysis should incorporate the way delta, gamma, and vega are normally calculated by practitioners (i.e., by substituting the implied volatility into the Black–Scholes–Merton formulas).

We have assumed that all the options traded with clients are “plain vanilla” European options. Further research could test how well our results carry over to exotic options. Our results show that an objective function involving a trade-off between mean and standard deviation works reasonably well when only plain vanilla options are traded. Preliminary results we have produced for barrier options show that hedging strategies produced using this objective function give quite different results from objective functions that focus on losses. This is because, when the underlying asset price is close to the barrier, the probability distribution of the derivative's price changes between hedge rebalancing is non-symmetrical.

We have used a fairly simple setup where a trade in at-the-money option with a certain maturity is used for hedging each day. In practice, only options with particular strike prices and times to maturity are available for trading on any given day. The RL approach must be used in conjunction with an algorithm for selecting from available options the one that is used for hedging each day. It is likely that a trader will wish to experiment with different algorithms.^11^ An extension to the analysis would be to assume that two options are available for hedging. The analysis is similar to that we have described but more computationally time-consuming.

In practice, the time horizon used (30 days in our tests) is likely to depend on the metrics used to assess the performance of the trader. A single analysis could be carried out at the beginning of a relevant period with the derived action as a function of the state variables being used throughout the period. Alternatively, the analysis could be updated periodically to reflect new information on the asset price process and the arrival of client orders.

As we wanted to focus on gamma and vega hedging, we assumed no transaction costs for delta hedging. The transaction costs associated with trading the underlying asset are almost invariably much less than those associated with trading options on the asset and tests that we have carried out suggest that the change in the optimal strategy arising from including these costs is not substantial. We also assumed no bid-ask spread on client options. Practitioners are likely to find it attractive to extend the analysis to incorporate both this and the transaction costs on the underlying asset so that a probability distribution for trading profitability is obtained. This can be done fairly easily.

For the sake of simplicity, we assumed proportional transaction costs throughout our experiments. Other tests could involve transaction costs that are convex or “fixed plus variable”. The bid-ask spread, which determines the transaction costs, could, with some increase in complexity, be assumed to be stochastic. The robustness tests that we have carried out are encouraging. However, more extensive tests where the true process followed by the asset price is quite different from the assumed process could be carried out.

The economics of trading derivatives is an important concern to dealers. In the example we considered, we have shown that the profits on trades more than cover the costs of hedging. It would be interesting to carry out further tests to see how this result depends on the average number of client orders per day and the transaction costs.

## Data availability statement

Publicly available datasets were analyzed in this study. This data can be found here: rotmanfinhub/gamma-vega-rl-hedging (github.com).

## Author contributions

All authors listed have made a substantial, direct, and intellectual contribution to the work and approved it for publication.

## Footnotes

^1^Trading rooms are generally organized so that responsibility for all derivatives dependent on a particular underlying asset is assigned to a single trader.

^2^At-the-money options are efficient hedging instruments because they have a large gamma and vega compared with similar maturity options that are significantly in- or out-of-the-money.

^3^We use D4PG with a single agent in all experiments since the distributed version is not necessary.

^4^This can be done by choosing an appropriate activation function in the last layer of the neural network that models the agent's action.

^5^As mentioned, we assume that delta hedging can be carried out at no cost. Our γ = 1 assumption is equivalent to assuming no funding costs and is not unreasonable given the short (30 day) time horizon considered.

^6^For the rest of the article, *S* refers to the asset price rather than the state.

^7^The general SABR model is (in a risk-neutral world) $dF=\sigma F^{\beta }dz_{1}$ with *dσ* = *vσdz*_2_ where *F* is the forward price of the asset for a particular maturity. We set β = 1 and assume that the volatility, σ, applies to the evolution of all forward prices. When the forward contract matures at time *T*, *S* = *Fe*^−(*r*−*q*)^^(*T*−*t*)^ so that *S* follows the process indicated.

^8^Gains are regarded as negative losses. When gamma and vega are hedged, *m* is positive because we do not take into account of the profit from the client options being hedged.

^9^The results presented are for *r* = *q* = μ = 0. We tried a range of other values for these parameters and the results were similar to those presented.

^10^We obtain good results in almost all our experiments, but there is no theory guaranteeing convergence to the optimal strategy.

^11^In our tests using the SABR model, Table 3 suggest that for transaction costs of 0.5 and 1% the longer maturity option is preferable, but for a transaction cost of 2% the shorter maturity option produces better results.

## References

[B1] Barth-Maron G. Hoffman M. W. Budden D. Dabney W. Horgan D. Tb D. (2018). Distributed distributional deterministic policy gradients. arXiv. 1804.08617.

[B2] Bellemare M. G. Dabney W. Munos R. (2017). “A distributional perspective on reinforcement learning,” in ICML'17 in proceedings of the 34th international conference on machine learning, vol. 70, 449–458.36121959

[B3] Black F. Scholes M. (1973). The pricing of options and corporate liabilities. J. Polit. Econ. 81, 637–659. 10.1086/26006210180222

[B4] Buehler H. Gonon L. Teichmann J. Wood B. (2019). Deep hedging. Quant. Finan. 19, 1271–1291. 10.1080/14697688.2019.1571683

[B5] Cao J. Chen J. Hull J. Poulos Z. (2021). Deep hedging of derivatives using reinforcement learning. J. Finan. Data Sci. Winter 3, 10–27. 10.3905/jfds.2020.1.052

[B6] Dabney W. Rowland M. Bellemare M. G. Munos R. (2018). “Distributional reinforcement learning with quantile regression,” in Proceedings of the AAAI Conference on Artificial Intelligence, 32, 2892–2901.36331649

[B7] Francois P. Galarneau-Vincent R. Gauthier G. Godin F. (2023). “Joint dynamics for the underlying asset and its implied volatility surface: a new methodology for option risk management,” in Working Paper. ssrn: 4319972.

[B8] Hagan P. Kumar D. Lesniewski A. S. Woodward D. E. (2002). Managing Smile Risk. Wilmott, 84–108.

[B9] Halperin I. (2017). QLBS: Q-learner in the Black-Scholes (-Merton) worlds. arXiv. 10.2139/ssrn.3087076

[B10] Kolm P. N. Ritter G. (2019). Dynamic replication and hedging: a reinforcement learning approach. J. Finan. Data Sci. Winter 2, 159–171. 10.3905/jfds.2019.1.1.159

[B11] Merton R. C. (1973). Theory of rational option pricing. Bell J. Econ. Manag. Sci. 4, 141–183. 10.2307/3003143

[B12] Tamar A. Castro D. D. Mannor S. (2016). Learning the variance of the reward-to-go. J. Mach. Learn. 17, 1–36.

